# SELective defunctioning Stoma Approach in low anterior resection for rectal cancer (SELSA): Protocol for a prospective study with a nested randomized clinical trial investigating stoma‐free survival without major LARS following total mesorectal excision

**DOI:** 10.1111/codi.70009

**Published:** 2025-01-30

**Authors:** Martin Rutegård, Marcus Lindsköld, Fredrik Jörgren, Kalle Landerholm, Peter Matthiessen, Håvard Mjørud Forsmo, Jennifer Park, Jacob Rosenberg, Johannes Schultz, Lars T. Seeberg, Josefin Segelman, Pamela Buchwald

**Affiliations:** ^1^ Department of Diagnostics and Intervention, Surgery Umeå University Umeå Sweden; ^2^ Department of Clinical Sciences Malmö Lund University Malmö Sweden; ^3^ Department of Surgery Ryhov County Hospital Jönköping Sweden; ^4^ Department of Biomedical and Clinical Sciences Linköping University Linköping Sweden; ^5^ Department of Surgery, Faculty of Medicine and Health Sciences Örebro University Örebro Sweden; ^6^ Department of Gastrointestinal Surgery Haukeland University Hospital Bergen Norway; ^7^ Department of Clinical Medicine University of Bergen Bergen Norway; ^8^ Department of Surgery Region Västra Götaland, Sahlgrenska University Hospital Östra Gothenburg Sweden; ^9^ Institute of Clinical Sciences, Sahlgrenska Academy, Gothenburg University Gothenburg Sweden; ^10^ Department of Surgery, Herlev Hospital University of Copenhagen Copenhagen Denmark; ^11^ Department of Paediatric and Gastrointestinal Surgery Oslo University Hospital Oslo Norway; ^12^ Institute of Clinical Medicine, University of Oslo Oslo Norway; ^13^ Department of Gastrointestinal Surgery Akershus University Hospital Lørenskog Norway; ^14^ Department of Gastrointestinal Surgery Vestfold Hospital Trust Tønsberg Norway; ^15^ Department of Surgery Ersta Hospital Stockholm Sweden; ^16^ Department of Molecular Medicine and Surgery Karolinska Institutet Stockholm Sweden

**Keywords:** anastomotic leakage, diverting, rectal cancer, stoma, TME

## Abstract

**Aim:**

Accumulated data suggest that routine use of defunctioning stoma in low anterior resection for rectal cancer may cause kidney injury, bowel dysfunction and a higher risk of permanent stomas. We aim to study whether avoidance of a diverting stoma in selected patients is safe and reduces adverse consequences.

**Methods:**

SELSA is a multicentre international prospective observational study nesting an open‐label randomized clinical trial. All patients with primary rectal cancer planned for low anterior resection are eligible. Patients operated with curative intent, aged <80 years, with an American Society of Anaesthesiologists' fitness grade I or II, and a low predicted risk of anastomotic leakage are eligible to 1:1 randomization between no defunctioning stoma (experimental arm) or a defunctioning stoma (control arm). The primary outcome is the composite measure of 2‐year stoma‐free survival without major low anterior resection syndrome (LARS). Secondary outcomes include anastomotic leakage, postoperative mortality, reinterventions, stoma‐related complications, quality of life measures, LARS score, and permanent stoma rate. To be able to state superiority of any study arm regarding the main outcome, with 90% statistical power and assuming 25% attrition, we aim to enrol 212 patients. Patient inclusion will commence in the autumn of 2024.

**Conclusion:**

The SELSA study is investigating a tailored approach to defunctioning stoma use in low anterior resection for rectal cancer in relation to the risk of anastomotic leakage. Our hypothesis is that long‐term effects will favour the selective approach, enabling some patients to avoid a defunctioning stoma.

**Trial registration:**

Swedish Ethical Review Authority approval (2023–04347‐01, 2024–02418‐02 and 2024–03622‐02), Regional Ethics Committee Denmark (H‐24014463), and ClinicalTrials.gov (NCT06214988).

## INTRODUCTION

Low anterior resection, comprising a total mesorectal excision (TME) and colorectal anastomosis, is the main curative option for mid and low rectal cancers located above the anal canal. Anastomotic leakage is a common complication, amounting to 10% within 30 days and 20% within the two first postoperative years in population‐based studies [1, 2]. A non‐restorative operation such as the Hartmann's procedure negates the risk of anastomotic leakage, but many patients have a strong desire to preserve bowel continuity [5]. Moreover, permanent stomas are not without short‐ and long‐term morbidity [6]. Nevertheless, anastomotic leakage carries the risk of both mortality and long‐term morbidity, including impaired bowel function or a permanent stoma [2, 3, 4]. A temporary defunctioning stoma aims to prevent or at least mitigate the immediate consequences of anastomotic leakage and is still considered standard of care [7]. However, in reality 18%–21% of these stomas are either never reversed or are converted to permanent colostomies within 2 years of resection surgery [8, 9]. Moreover, loop ileostomies may confer high output, which can cause dehydration and even acute kidney injury, especially when not reversed in a timely fashion [10, 11]. It is also likely that defunctioning stomas increase the risk of severe low anterior resection syndrome (LARS), a related cluster of bowel dysfunction symptoms that seems to stabilize only after 18 months [12, 13].

As more data on long‐term negative consequences of defunctioning stomas are emerging, the question is whether short‐term advantages outweigh long‐term stoma‐related morbidity and mortality. In recent years, Dutch nationwide data demonstrated a decrease in the use of defunctioning stomas down to 30% along with a concurrent increase in leak and reintervention rates, whereas postoperative mortality has decreased simultaneously [14]. Attempts to risk‐stratify patients and tailor stoma use have not been widely successful, but some centres have reported a selective use of stomas without obvious adverse consequences [15]. There is one ongoing French randomized clinical trial, GRECCAR‐17, investigating routine loop ileostomy (control arm) versus tailored loop ileostomy (intervention arm) based on a leakage risk prediction score [16].

The present study aims to evaluate a selective approach to bowel diversion after low anterior resection, avoiding defunctioning stomas in low‐risk patients, based on the predicted risk of anastomotic leakage as well as the patients' perceived ability to tolerate an anastomotic leakage. The main outcome consists of stoma‐free survival without major bowel dysfunction 2 years after resection surgery.

## METHODS

### Trial design and setting

The SELective defunctioning Stoma Approach in low anterior resection for rectal cancer (SELSA) trial is a pragmatic multicentre Scandinavian prospective observational study including a nested non‐blinded randomized clinical superiority trial with two parallel groups. Patients are block randomized intraoperatively within a REDCap database using permuted blocks of sizes 2 or 4 and a 1:1 allocation, stratified for centre and radiotherapy. The study protocol was drafted in accordance with the SPIRIT 2013 statement (Standard Protocol Items: Recommendations for Interventional Trials) [17]. The trial registration data set is detailed in Table S1.

### Eligibility criteria

**FIGURE 1:**
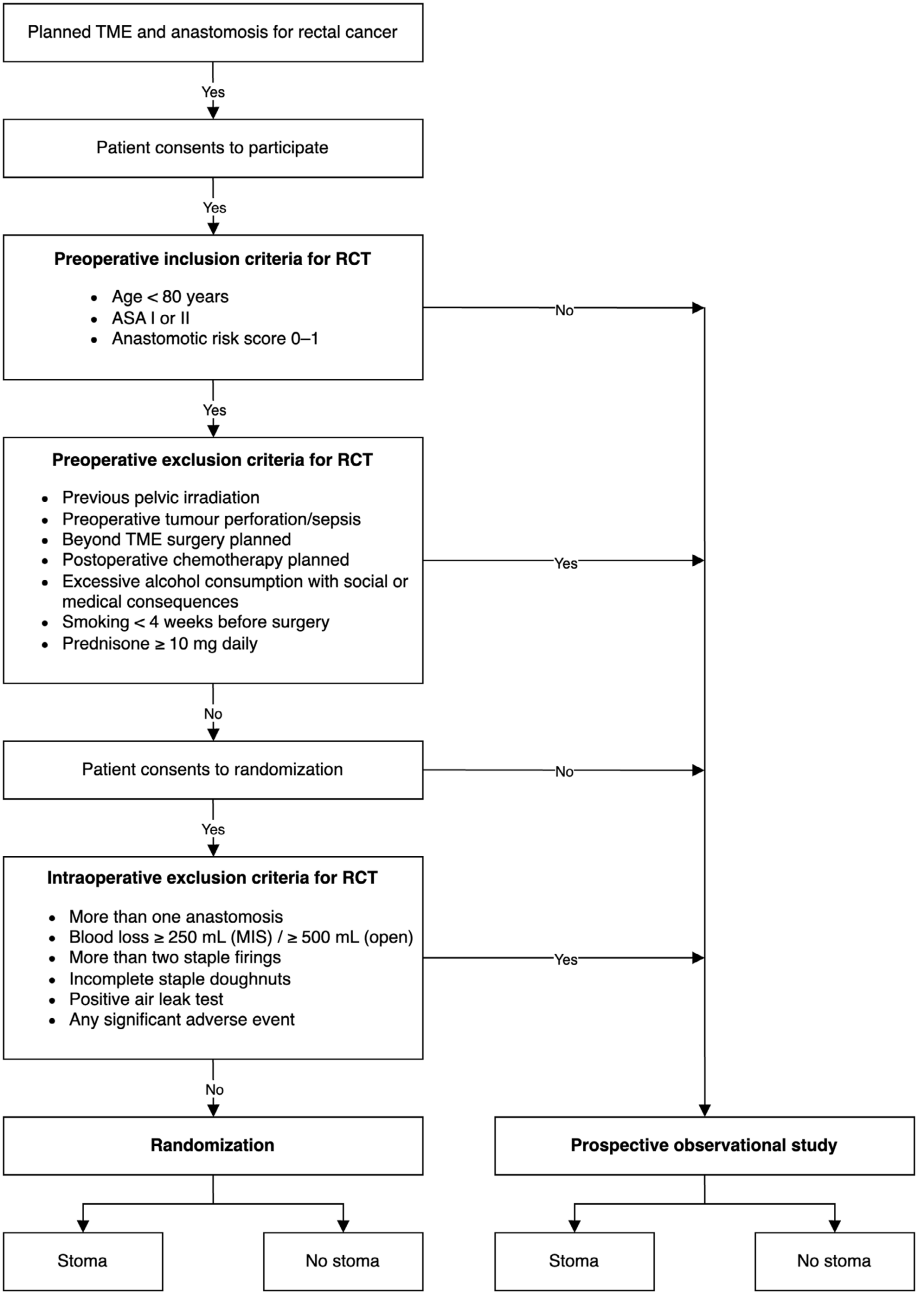
SELSA study flow chart. ASA, American Society of Anaesthesiologists' fitness grade; LARS, low anterior resection syndrome; MIS, minimally invasive surgery; TME, total mesorectal excision.

A complete list of eligibility criteria is displayed in Table 1. All adult (≥18 years old) patients with rectal cancer (inferior tumour margin ≤15 cm from the anal verge, measured with rigid sigmoidoscopy) planned for a TME procedure with curative intent, and an anastomosis, are eligible for inclusion. Patients undergoing partial mesorectal excision are ineligible. Patients undergoing emergency rectal resection, currently pregnant or breastfeeding or with insufficient command of the study languages will be excluded. Additional inclusion criteria for the nested randomized trial are <80 years of age, ASA fitness grade I–II, anastomotic leak risk score (described below) of 0–1 and willingness to be randomized. Additional exclusion criteria for the randomized part of the study are previous pelvic irradiation, preoperative tumour perforation or pelvic sepsis, a surgical procedure beyond TME and/or concurrent resection of another organ, corticosteroid treatment, planned postoperative chemotherapy (regardless of histopathological findings), active smoking and excessive alcohol consumption. Intraoperative exclusion criteria for the randomized part are: ≥3 staple firings for rectal transection, high intraoperative blood loss (≥250 mL for minimally invasive and ≥500 mL for open surgery), ≥2 intraabdominal anastomosis performed, incomplete stapler doughnuts, positive air‐leak test, any significant intraoperative adverse event as determined by the operating surgeon, and TME with anastomosis ultimately not performed. The SELSA study flow chart is demonstrated in Figure 1.

**TABLE 1:** Eligibility criteria for the SELSA study including an observational study and a nested randomized controlled trial.

Eligibility criteria
Inclusion criteria
Adult patients with rectal cancer planned for a curative low anterior resection with anastomosis by TME with any surgical approach
Exclusion criteria
Insufficient command of Swedish, Norwegian, Danish, or English to understand questionnaires or consent Emergency rectal resection (tumour resection due to bowel obstruction, perforation, etc.) Pregnancy or breastfeeding
Additional inclusion criteria for randomized part of the study
Patients aged <80 years Patients with American Society of Anaesthesiologists' fitness grade I or II as determined by the anaesthesiologist or the surgeon Anastomotic leak risk score of 0–1 Willingness to be randomized
Additional exclusion criteria for randomized part of the study
Previous pelvic irradiation (due to e.g., gynaecological or urological cancer) Preoperative tumour perforation or pelvic sepsis Beyond TME surgery and/or concurrent resection of other organs Concurrent corticosteroid treatment (prednisone‐equivalent dosage ≥10 mg daily) Planned postoperative chemotherapy Smoking not completely ceased 4 weeks before surgery Excessive alcohol consumption with social and medical consequences (as judged by the surgeon in charge)
Intraoperative exclusion criteria for randomized part of the study
More than two staple firings for rectal transection Intraoperative blood loss ≥250 mL for minimally invasive surgery Intraoperative blood loss ≥500 mL for open or converted surgery More than one intra‐abdominal anastomosis performed Incomplete stapler doughnuts Positive air‐leak test Any significant intraoperative adverse event at the discretion of the operating surgeon (e.g., ureterotomy, bowel or tumour perforation, major medical event such as pulmonary embolism or cardiac arrhythmia) TME with anastomosis ultimately not done

Abbreviation: TME, total mesorectal excision.

### Anastomotic risk score

To test the intervention in a low‐risk group, with a predicted anastomotic leakage risk of <10%, a prediction model was developed using data from the CRCBaSe database [18]. This database integrates the Swedish Colorectal Cancer Registry (SCRCR) with several national patient registries containing pertinent data such as comorbidities and prescribed drugs. Inclusion and exclusion criteria in the modelling cohort were closely aligned with the eligibility criteria of the SELSA trial. The development of this model incorporated established risk factors for anastomotic leakage including male sex, elevated body mass index (BMI), preoperative radiotherapy, diabetes and cardiovascular disease. The resulting model was internally validated with a negative predictive value of 94.7%. Subsequent validation in an independent multicentric cohort with chart‐reviewed anastomotic leakage data [19] demonstrated a corresponding negative predictive value of 89.9% (submitted, Rutegård 2024). This risk score model, ultimately comprising the variables male sex, BMI >30, and preoperative radiotherapy, will be used to stratify the risk of anastomotic leakage (Table 2).

**TABLE 2:** Risk prediction modelling where male sex, BMI >30, and radiotherapy each denote one point.

Risk score	SCRCR leak rate (%)	RectoLeak leak rate (%)
0	2.4	2.8
1	5.4	17.7
≥2	11.1	23.7

*Note*: Leak rates for different risk score groups are described, comparing registry data in CRCBaSe to chart‐reviewed multicentre cohort data [19].

Abbreviations: BMI, body mass index; SCRCR, Swedish Colorectal Cancer Registry.

### Recruitment

All Scandinavian hospitals that perform low anterior resections can participate if they have the resources to perform necessary trial measurements, provide timely reintervention for suspected anastomotic leakage, and have at least one responsible local investigator. Eligible patients will be identified at multidisciplinary team meetings and will be asked to participate at preoperative outpatient clinics.

### Interventions in the nested randomized trial

In the experimental arm, no defunctioning stoma is formed. In the control arm, a defunctioning stoma is constructed at a preoperatively marked stoma site. A loop ileostomy is fashioned using an ileal loop close to the ileocaecal valve, while a loop colostomy can be derived from either the transverse or a redundant left colon.

### Preoperative measures, low anterior resection, and postoperative surveillance

At least one stoma site is marked preoperatively by a specialized nurse. Mechanical bowel preparation and preoperative antimicrobial prophylaxis is employed according to local routines. Patients will be managed according to the principles outlined in the Enhanced Recovery After Surgery (ERAS) guidelines for rectal surgery [20].

Any surgical approach is allowed, whether open or minimally invasive (robotic‐assisted, conventional laparoscopy, or transanal TME). Splenic flexure mobilization, left colic artery preservation, use of descending or sigmoid colon as conduit, or anastomotic configuration is at the discretion of the operating surgeon. TME should be conducted according to the principles of dissection in embryological planes, below the tapering mesorectum down to the bare rectal muscle tube [21]. Adjuncts such as near infrared indocyanine green assessment are allowed, but only to influence decision‐making prior to construction of the anastomosis. The anastomosis, situated at the pelvic floor following a complete TME resection, can be performed with a double‐ or single‐stapling technique, allowing for modifications such as oversewing in accordance with the surgeon's standard practice. Handsewn coloanal anastomoses are not permitted. After construction of anastomosis, the integrity of the doughnuts should be evaluated, and an air‐leak test should be performed. Agreement with the anaesthesia team should be sought to determine whether the intraoperative exclusion criteria listed above apply. If the patient is still deemed eligible, the patient will be randomized. Patients who do not meet the randomization criteria will receive either a defunctioning stoma and take part in the observational study or no anastomosis at all, as determined by the surgical team. Drains will be used at the discretion of the operating surgeon but are generally discouraged. Anastomotic height is measured by digital rectal examination or endoscopy and noted. Anastomotic leakages will be handled in a timely fashion at the discretion of the surgical team, with an appropriate intervention according to the severity of the leakage.

Defunctioning stoma reversal will be scheduled and performed according to local routines, typically 3–6 months after index surgery if no leakage or other complications have occurred. In cases requiring adjuvant chemotherapy, stoma reversal is planned after completion of treatment, usually 9–12 months after the index surgery. The integrity of the anastomosis will be determined by flexible sigmoidoscopy and/or computed tomography (CT) with water soluble contrast enema 1–3 months and 12 months postoperatively.

Administration of neoadjuvant and adjuvant radio‐ and chemotherapy, as well as other oncological treatment, is at the discretion of the local multidisciplinary team meeting. Oncological follow‐up adheres to national guidelines. This includes, at a minimum, CT of the chest, abdomen and pelvis and carcinoembryonic antigen (CEA) at 1 and 3 years, and continued colonoscopy surveillance per national protocols. The management of any recurrences will be decided at the local multidisciplinary team meeting and will be registered via national quality registries as well as in an electronic case report form (eCRF).

### Outcome assessment

The primary outcome is a composite outcome: stoma‐free survival without major LARS at 2 years, reflecting a functionally appropriate outcome after low anterior resection for rectal cancer. The summary measure is the proportion of patients fulfilling this composite outcome 2 years after the anterior resection: alive, no stoma and no major LARS (LARS score < 30). This time point is chosen as most stomas are considered permanent 2 years after surgery, whereas bowel function has stabilized in those without a stoma in situ [12]. The secondary outcomes, along with measurement variables, analysis metrics, aggregation methods and measurement time points, are presented in Table 3. Of note, all participating patients will have the same outcome assessment, regardless of eligibility for randomization.

**TABLE 3:** Secondary outcomes in the SELSA trial.

Secondary outcome	Measurement variable	Analysis metric	Summary measure	Time point (months)
Anastomotic leakage	ISREC grading	Final value	Proportion	1, 3, 12, 24
Complications	Clavien‐Dindo grade	Final value	Proportion	1, 3, 12, 24
Length of hospital stay	Total days in‐hospital	Final value	Median	At discharge
Postoperative mortality	Clinical assessment categorization	Final value	Proportion	3
Major LARS	LARS domain score	Change from baseline and spot measure	Proportion	12, 24
Quality of life	EORTC‐C30 domain scores	Change from baseline and spot measure	Median	12, 24
Quality of life	EORTC‐CR29 domain scores	Change from baseline and spot measure	Median	12, 24
QoR15	Total QoR15 score	Change from baseline	Median	1
Adjuvant chemotherapy for high‐risk patients	Clinical assessment categorization	Final value	Proportion	12
Renal function	Creatinine (mg/L)	Change from baseline and spot measure	Median	12, 24
Stay out of hospital	Total days out of hospital and alive	Final value	Median	24
Stoma in situ	Clinical assessment categorization	Final value	Proportion	12, 24
Recurrence (locoregional and distant)	Clinical assessment categorization	Final value	Proportion	36, 60
Overall survival	Clinical assessment categorization	Final value	Proportion	36, 60

Abbreviations: EORTC, European Organization for Research and Treatment of Cancer; ISREC, International Study Group for Rectal Cancer; LARS, low anterior resection score; QoR, quality of recovery.

### Measures of complications

Anastomotic leakage is defined according to the International Study Group of REctal Cancer (ISREC) consensus definition [22], which states that any communication between intra‐ and extraluminal compartments is considered a leakage. This includes fistulae as well as isolated pelvic abscesses, even without evidence of direct communication. Depending on need for reintervention, anastomotic leakages will be categorized into three levels (A: no active intervention; B: any intervention except relaparotomy or relaparoscopy; C: relaparotomy or relaparoscopy required) and will be characterized by day of diagnosis, leak grade, type of leak, modalities used for diagnosis and management of the leak itself, including subsequent reoperations and other measures. This will be included in an eCRF and will be registered at several time points. In particular, when postoperative examinations (1–3 months and 12 months) using flexible sigmoidoscopy and/or contrast enema (with or without CT) are required in order to detect asymptomatic anastomotic leakages. Measures of complications will be registered and ascertained using an eCRF at all time points. Study‐specific variables include stoma‐related complications (high‐output stoma, admission for dehydration, kidney injury, stoma retraction, stoma prolapse, symptomatic parastomal hernia, peristomal skin irritation, stoma leaks, or complications of stoma reversal).

### Sample size

The sample size for the whole study is based on power calculations for the nested randomized superiority trial. The sample size is calculated with the following assumptions:

- Proportion of patients with stoma‐free survival without major LARS is estimated at 65% in the experimental no stoma group
- Proportion of patients with stoma‐free survival without major LARS is estimated at 40% in the control stoma group
- The loss of data due to, for example, non‐adherence to study protocol, is anticipated to be 25%

The null hypothesis states no difference in stoma‐free survival and the alternative hypothesis is that there is a difference. A total of 158 randomized patients (79 in each arm) are needed to detect a difference in proportion of 25% units (65% vs. 40%) between the group with 90% statistical power and a significance level of 5%. Considering attrition and missing data, we aim to include 212 participants.

The first 100 included patients will be regarded as a feasibility phase. After inclusion of this feasibility cohort, assumptions will be checked descriptively, without breaking the randomization code. This will then inform potential adjustments to the sample size.

### Statistical analysis

A detailed statistical analysis plan will be developed (and locked) prior to data analysis. All primary analyses in the randomized part of the study will be performed according to the intention‐to‐treat (ITT) principle, that is, participants will be analysed in the treatment group they were allocated to irrespective of the treatment they eventually received. The ITT population will consist of all randomized patients. All statistical tests of safety will be conducted with a two‐sided test, using an alpha level of 0.05.

### Primary outcome analysis

The main analysis in this trial consists of a comparison of the proportion of alive stoma‐free patients without major LARS at 2 years after surgery in trial arm. The *χ* ^2^ test will be used in an ITT analysis. A sensitivity analysis will be conducted, with adjustment for centre as well as preoperative variables indicating a high risk of anastomotic leakage and permanent stoma, such as sex, age, ASA fitness grade, radiotherapy and clinical tumour stage. These adjustments, using binomial regression, are done to alleviate the impact of chance confounding; effect size will be estimated by relative risk ratios and absolute risk differences with 95% confidence intervals will be produced. As sensitivity analyses, non‐inferiority (assuming a non‐inferiority margin of 10%) and Bayesian approaches will be explored. Missing data will be handled with multiple imputation by chained equations.

### Secondary analyses

Comparison between proportions in study arms will be made with the *χ* ^2^ test or the Fisher's exact test, while comparisons of continuous variables will be made with the student's *t*‐test or Mann–Whitney U test. The pertinent outcomes and time points are outlined in Table 3. The following exploratory secondary outcome comparisons between study arms will be conducted:

- Anastomotic leakage
- Leakage grade
- Stoma‐related complications
- Length of hospital stay
- Postoperative mortality at 90 days
- Incidence of major LARS
- Quality of life measured with QLQ‐C30 and QLQ‐CR29
- Postoperative recovery measured with QoR15
- Adjuvant chemotherapy receipt in patients deemed high‐risk for recurrence
- Renal function
- Accumulated hospital stay at 90 days
- Stoma in situ at 24 months after surgery

### Patient discontinuations

Reasons for discontinuations in the study will be compared between the two treatment groups and described for the non‐randomized patients. Tables and/or a CONSORT diagram will reveal the number and proportion of patients who have completed the study as well as patients who have discontinued, grouped by reasons for discontinuation [23].

### Missing data

Every effort will be made to ensure that missing data is kept to a minimum. As the primary outcome is dependent on several parameters, one of which is derived from the LARS questionnaire, imputation is planned to compensate for any missing data for the 24‐month assessment, using available data on perioperative and functional information from other time points.

### Registry‐based comparison

All patients participating in the SELSA trial are also included in the prospectively collected quality registry data from Sweden (SCRCR), Denmark (DCCG), and Norway (CRN‐CR). These data are important to provide information about the representativeness of the trial population. Further, the data can be used to provide external validation of effectiveness, where defunctioning stoma use or no use is treated as exposure and anastomotic leakage as well as stoma‐free survival can be seen as outcome.

### Trial management

SELSA will be led by the principal investigators (PI)s (MR and PB). A trial management group comprising the PIs, trial research nurses, coapplicants and statistician has been created. A data monitoring committee (DMC) consisting of experienced colorectal surgeons likewise senior professors of surgery, statistician and patient representative has been appointed. Centres will be asked annually to report unforeseen adverse events and problems. The DMC will have access to study data to carry out its tasks. If severe adverse events occur, the trial steering committee (PIs and the DMC) has the responsibility to decide whether the study can continue or should be stopped after a safety analysis – such a safety analysis will be executed after recruitment and 90‐day follow‐up of 50 patients. A higher reoperation rate is anticipated in the experimental group, where earlier data suggest that this may be up to three times as likely [7]; however, this is not unsafe on its own, but rather the study termination criterion will be substantial differences in Clavien‐Dindo ≥IV (organ failure requiring admission to intensive care unit [24]). All safety analyses will be performed based on the ITT population.

### Quality assurance

To ensure accurate and reliable data the study administration will do the following:

1. Instigate start‐up training and meetings with investigators
2. Be available for consultation
3. Have regular communication to investigators with data on recruitment
4. Conduct quality review of the database (a monitor will visit participating centres)

The study will monitor study interventions while the trial is underway. Nevertheless, individual surgical teams and centres are responsible for adherence to the protocol.

### Ethics and dissemination

National ethical approval has been or will be granted by the relevant authorities, where laymen are involved. Informed consent is required from all participating patients. The results will be disseminated through patient associations, popular science, the broader medical community and conventional scientific channels.

### Patient involvement

This study protocol has been created in conjunction with national and international patient representatives to reflect outcomes that are important to patients. The risks to the participants, associated with the study, are deemed tolerable by patient representatives in relation to possible gains.

### Translational substudy

Participating centres are invited to collect preoperative blood samples from all patients for later molecular analysis of predictors for anastomotic leakage. Previous research has indicated that chemokines C‐X‐C motif ligand 6 and C‐C motif ligand 11 might be strong predictors of leakage in patients with rectal cancer, but these findings require external validation [25]. Area under the curve with receiver operating characteristics will be derived for each protein as well as selected combinations. Thresholds to optimize subsequent diagnosis of leakage will be calculated. Other inflammation‐related proteins will be explored (Table S2).

## DISCUSSION

The SELSA trial will be one of the first trials to compare selective to routine use of defunctioning stoma following TME for rectal cancer. The patient‐centred composite primary endpoint of stoma‐free survival at 2 years without major LARS ensures that both bowel function and safety are considered.

Although reducing the risk of early reinterventions for anastomotic leakage, a defunctioning stoma carries the risk of considerable postoperative morbidity, which sometimes could be long‐term and even lifelong. In this trial, all stoma‐related complications will be recorded and graded according to the Clavien‐Dindo classification over a 2‐year period. Consequently, the trial will prospectively collect high quality data in patients undergoing TME with or without a defunctioning stoma with the aim to provide scientific evidence in the management of patients with rectal cancer subjected to low anterior resection.

Patient‐reported outcome measures such as quality of life and functional outcomes require greater focus in rectal cancer research. Here, care has been taken to provide a protocol that is acceptable to patients regarding additional investigations including blood tests and integrity check of the anastomosis. We have chosen to include QLQ‐C30 and QLQ‐CR29 since these are well‐established questionnaires used in similar studies making the results comparable with e.g. GRECCAR‐17 [16]. We have also included a questionnaire (QoR15) measuring patient reported quality of recovery after surgery and anaesthesia on the advice of patient representatives [26].

A potential drawback of the study is the power calculation assumptions. These are based on a synthesis of the available data, where prior studies have not directly addressed our primary outcome stoma‐free survival without major LARS. Mortality rates were derived from Swedish audit data [27], whilst permanent stoma rates at 2 years have been reported to be 20% versus 9% in TME patients with and without a defunctioning stoma [9]. In addition, the occurrence of major LARS has been estimated at 57 versus 29% in patients with and without a defunctioning stoma, several years after surgery [13]. Amalgamating these data into the composite primary outcome is not straightforward, and there is a real possibility that, as a result, the trial could prove to be underpowered. The possibility to readjust the sample size after 100 included patients is an attempt to minimize this weakness. Another potential limitation could be the reliability of the anastomotic risk score, given the inherent complexity of predicting anastomotic leakage [28]. A minority of patients with a predicted leakage risk below our trial threshold of 10% may inevitably exhibit a higher risk. Nevertheless, the risk prediction model was developed and tested in accordance with contemporary guidelines [29].

Many centres have already implemented a selective approach for defunctioning stomas following TME. However, most Scandinavian centres still use defunctioning stomas routinely and the SELSA study group therefore considers the trial to be feasible. Furthermore, since the study contains an observational part, all patients undergoing TME for rectal cancer with anastomosis are eligible for inclusion, providing a benchmark for comparison to the randomized part of the study. The inclusion rate of all available patients will also be cross‐checked against Scandinavian national quality registries.

The exact causes of anastomotic leakage remain mostly uncharted territory, probably involving an interplay between surgeon and patient factors, including the inflammatory response and gut microbiota, resulting in failed healing. SELSA will include a translational part where baseline blood samples are collected from participating patients to further explore biomarkers for leakage. Our main aim is to validate previously reported inflammation‐related chemokines, but there will also be opportunities for future investigations [25].

In conclusion, the SELSA trial will investigate the impact of a patient‐tailored approach on defunctioning stomas on stoma‐free survival and major LARS following TME for rectal cancer. Patient risk stratification will be guided by a validated prediction score for anastomotic leakage. This study will provide evidence of patient‐reported outcomes and function in an era where long‐term survivorship is better recognized.

## AUTHOR CONTRIBUTIONS

**Martin Rutegård:** Conceptualization; investigation; writing – original draft; funding acquisition; methodology; visualization; writing – review and editing; project administration; data curation; resources; formal analysis; software. **Marcus Lindsköld:** Writing – review and editing; validation; resources; methodology. **Fredrik Jörgren:** Conceptualization; investigation; methodology; writing – review and editing; validation; resources. **Kalle Landerholm:** Conceptualization; investigation; methodology; visualization; writing – review and editing; software; validation; resources. **Peter Matthiessen:** Investigation; conceptualization; methodology; writing – review and editing; validation; resources. **Håvard Mjørud Forsmo:** Resources; conceptualization; investigation; methodology; validation. **Jennifer Park:** Resources; conceptualization; investigation; methodology; writing – review and editing; validation; project administration. **Jacob Rosenberg:** Conceptualization; investigation; project administration; resources; writing – review and editing; methodology; validation. **Johannes Schultz:** Conceptualization; investigation; methodology; validation; writing – review and editing; resources. **Lars T. Seeberg:** Conceptualization; investigation; resources; writing – review and editing; project administration; validation; methodology. **Josefin Segelman:** Conceptualization; investigation; writing – review and editing; methodology; validation; project administration; resources. **Pamela Buchwald:** Conceptualization; investigation; funding acquisition; writing – original draft; writing – review and editing; methodology; validation; project administration; resources; supervision.

## FUNDING INFORMATION

The trial has received financial support from the Swedish Cancer Society (233221 S) and the Swedish Research Council (VR 2023‐06400).

## CONFLICT OF INTEREST STATEMENT

The authors declare no conflicts of interest.

## TRIAL SPONSOR

Skåne University Hospital, Region Skåne, Sweden, is the study sponsor. The study sponsor has no authority over any study activity. The sponsor can be reached via www.skane.se/kontakt.

## PREVIOUS COMMUNICATIONS

The SELSA study protocol has been presented in abstract form at the Swedish Surgical Week in August 2023 (Örebro, Sweden), the Norwegian Surgical meeting in October 2023 (Oslo, Norway), and the annual European Society of Coloproctology meeting in September 2023 (Vilnius, Lithuania).

## PROTOCOL VERSION

Version 1.1, 27 September, 2024.

## Supporting information


Data S1.

Table S1.

Table S2.

## Data Availability

The trial steering and the data monitoring committees will have access to the final data set. Subject to steering committee approval, data can be shared upon reasonable request.
