# Supplementary material for: SELective defunctioning Stoma Approach in low anterior resection for rectal cancer (SELSA): Protocol for a prospective study with a nested randomized clinical trial investigating stoma‐free survival without major LARS following total mesorectal excision
Source: Colorectal Dis. 2025 Jan 30;27(2):e70009. doi: 10.1111/codi.70009 (PMC11780343; doi:10.1111/codi.70009)
Supplement: Supplementary file 2 — Table S1. [file CODI-27-0-s001.docx]

**Table S1.** SELSA trial data registration set.

| **Data category** | **Information** |
| --- | --- |
| Primary registry and trial identifying number | ClinicalTrials.gov NCT06214988 |
| Date of registration in primary registry | 22^nd^ of January, 2024 |
| Secondary identifying numbers | SELSA-2023-04347-01 |
| Source(s) of monetary or material support | Swedish Cancer Society, Swedish Research Council |
| Primary sponsor | Region Skåne |
| Secondary sponsor(s) |  |
| Contact for public queries | Caroline Nilsson (caroline.n.nilsson@skane.se) |
| Contact for scientific queries | Caroline Nilsson (caroline.n.nilsson@skane.se) |
| Public title | Selective Defunctioning Stoma in Low Anterior Resection for Rectal Cancer (SELSA) |
| Scientific title | SELective defunctioning Stoma Approach in low anterior resection for rectal cancer (SELSA): a prospective study with a nested randomized clinical trial investigating stoma-free survival without major LARS following total mesorectal excision |
| Countries of recruitment | Sweden, Denmark, Norway |
| Health condition(s) or problem(s) studied | Rectal cancer |
| Intervention(s) | Experimental arm: no defunctioning stoma |
|  | Control arm: defunctioning stoma |
| Key inclusion and exclusion criteria | Ages eligible for study: ≥18 years  Sexes eligible for study: both Accepts healthy volunteers: no |
|  | Inclusion criteria: patients planned for a low anterior resection by total mesorectal excision with curative intent |
|  | Exclusion criteria: age ≥80 years, American Society of Anesthesiologists’ grade ≥III, predicted leakage risk >10%, previous pelvic sepsis or irradiation, multivisceral surgery, corticosteroid treatment, active smoking, multiple staple firings, excessive blood loss, anastomotic problems |
| Study type | Interventional |
|  | Allocation: randomised, controlled, non-blinded, multicentre non-inferiority trial with two parallel groups |
|  | Primary purpose: reduce stoma permanence and bowel dysfunction |
|  | Phase III |
| Date of first enrolment | September 2024 |
| Target sample size | 212 |
| Recruitment status | Recruiting |
| Primary outcome(s) | Stoma-free survival without major low anterior resection syndrome two years after surgery |
| Key secondary outcomes | Anastomotic leakage, quality of life, stoma complications |
