# Supplementary material for: SELective defunctioning Stoma Approach in low anterior resection for rectal cancer (SELSA): Protocol for a prospective study with a nested randomized clinical trial investigating stoma‐free survival without major LARS following total mesorectal excision
Source: Colorectal Dis. 2025 Jan 30;27(2):e70009. doi: 10.1111/codi.70009 (PMC11780343; doi:10.1111/codi.70009)
Supplement: Supplementary file 3 — Table S2. [file CODI-27-0-s002.docx]

**Table S2.** Selected proteins of interest in the translational sub study on prediction of anastomotic leakage.

| Assay name | UniProt ID | Gene |
| --- | --- | --- |
| Eotaxin | P51671 | CCL11 (SCYA11) |
| Eukaryotic translation initiation factor 4E | P06730 | EIF4E (EIF4EL1 EIF4F) |
| C-C motif chemokine 8 | P80075 | CCL8 (MCP2 SCYA10 |
| C-X-C motif chemokine 11 | O14625 | CXCL11 (ITAC SCYB11 |
| Tumour necrosis factor ligand superfamily member 14 | O43557 | TNFSF14 (HVEML LIGHT) |
| Tumour necrosis factor receptor superfamily member 9 | Q07011 | TNFRSF9 (CD137 ILA) |
| Adenosine deaminase | P00813 | ADA (ADA1) |
| C-C motif chemokine 25 | O15444 | CCL25 (SCYA25 TECK) |
| STAM-binding protein | O95630 | STAMBP (AMSH) |
| Caspase-8 | Q14790 | CASP8 (MCH5) |
| Leukaemia inhibitory factor receptor | P42702 | LIFR (CD118) |
| Interleukin-6 | P05231 | IL6 (IFNB2) |
| C-X-C motif chemokine 6 | P80162 | CXCL6 (GCP2 SCYB6) |
| Interleukin-8 | P10145 | CXCL8 (IL8) |
| Vascular endothelial growth factor A | P15692 | VEGFA (VEGF) |
| Matrilysin | P09237 | MMP7 (MPSL1 PUMP1) |
| Growth-regulated alpha protein | P09341 | CXCL1 (GRO GRO1 |
| Interleukin-7 | P13232 | IL7 |
| C-C motif chemokine 20 | P78556 | CCL20 (LARC MIP3A |
| Hepatocyte growth factor | P14210 | HGF (HPTA) |
| Kit ligand | P21583 | KITLG (MGF SCF) |
